# Iris Cysts: Clinical Features, Imaging Findings, and Treatment Results

**DOI:** 10.4274/tjo.galenos.2019.20633

**Published:** 2020-03-05

**Authors:** Helin Ceren Köse, Kaan Gündüz, Melek Banu Hoşal

**Affiliations:** 1 Ankara University Faculty of Medicine, Department of Ophthalmology, Ankara, Türkiye

**Keywords:** Iris pigment epithelial cyst, siris stromal cyst, SS-OCT, SS-OCTA, UBM

## Abstract

**Objectives:**

To report the clinical and demographic characteristics, imaging findings, treatment results, and follow-up data of patients with iris cysts.

**Materials and Methods:**

The medical records of 37 patients with iris cysts were retrospectively analyzed. Ultrasound biomicroscopy (UBM), swept-source optical coherence tomography (SS-OCT), and SS-OCT angiography (SS-OCTA) were performed to examine the iris cysts.

**Results:**

The mean age of the patients was 34.4 years, ranging from 5 to 85 years. Twenty-four patients (65%) were female and 13 (35%) were male. Mean follow-up period was 21.3 months, ranging from 4 months to 8 years. Thirty-five (94.5%) of the cysts were classified as primary and 2 (4.5%) were classified as secondary. Thirty-one (83.7%) of the primary cysts were pigment epithelial and 4 were stromal. Primary iris pigment epithelial cysts were classified as peripheral in 26 patients (72.2%), midzonal in 4 (11.1%), and dislodged in 1 (2.7%). Stromal cysts were classified as acquired in 3 patients (8.1%) and congenital in 1 patient (2.7%). Secondary iris cysts were caused by perforating eye injury. UBM could visualize both the anterior and posterior surfaces of the cysts (26 patients). Anterior segment SSOCT could visualize the anterior but not the posterior surface of the cysts (4 patients). Iris cysts did not display intrinsic vascularity on SS-OCTA (4 patients). All pigment epithelial cysts were managed by observation. Of the 4 primary stromal cysts, 3 were managed by surgical excision and 1 by observation. Two secondary cysts required surgical removal.

**Conclusion:**

Pigment epithelial cysts generally remain stable without need for treatment. However, iris stromal cysts frequently require surgical intervention. UBM and SS-OCT were valuable in the diagnosis of iris cysts. On UBM, iris cysts appear with a thin, hyperechoic wall with hypoechoic internal content. Iris cysts did not have intrinsic vascularity on anterior segment SS-OCTA.

## Introduction

Iris tumors are categorized as cystic and noncystic (solid) lesions. In a study examining 3,680 iris tumors, 21% were reported to be cystic (n=718) and 79% noncystic (n=2.733).^1^ Cystic lesions originate from the iris pigment epithelium (IPE) or iris stroma.^2,3^

In a classification made by Shields^4^ in 1981, iris cysts are grouped as primary and secondary according to their etiology, then divided into subgroups based on their tissue or origin. According to this classification, primary IPE cysts are the most common in clinical practice. Most IPE cysts are asymptomatic.^4,5^ Primary cysts are usually of neuroepithelial origin and rarely lead to complications. Primary IPE cysts have 4 subtypes based on their location: pupillary, midzonal, peripheral, and free-floating/dislodged.^4,5,6^ Secondary cysts, on the other hand, develop due to causes such as implantation after penetrating or surgical trauma, intraocular tumors, prolonged use of prostaglandins and miotics, or parasitic infections such as ocular cysticercosis.^6,7,8,9,10^ They may lead to complications such as decreased visual acuity, secondary glaucoma, corneal edema, or uveitis.

Stromal iris cysts can be congenital or acquired.^11,12^ They can remain dormant for years or suddenly grow and rupture, leading to secondary glaucoma and corneal decompensation. Although stromal cysts can occasionally regress spontaneously, most require treatment by needle aspiration or surgical excision.^12,13^

Slit-lamp examination, anterior segment (AS) optical coherence tomography (OCT), and ultrasound biomicroscopy (UBM) are used in the differential diagnosis of iris cysts.^14^ These techniques are important in ruling out solid tumors such as nevus or melanoma.

The aim of this study was to discuss the demographic and clinical features, treatment, and follow-up results of patients with iris cysts.

## Materials and Methods

The records of 37 seven patients who were treated and followed for iris cysts in the ocular oncology unit of the Medical Faculty of Ankara University Ophthalmology Department between January 2007 and January 2019 were retrospectively reviewed. The type, etiology, location, and size of the cyst, treatments applied, recurrence status, and outcome at final follow-up were analyzed. Cyst size was measured with UBM in 26 patients. Four patients underwent AS swept-source OCT (SS-OCT) (Topcon, Tokyo, Japan) and SS-OCT angiography (SS-OCTA) imaging. Three of the patients with stromal cysts underwent partial lamellar sclerouvectomy (PLSU) and 2 underwent surgical resection via limbal incision.

The study was conducted in accordance with the principles of the Declaration of Helsinki. Approval was obtained from our institutional ethics committee and informed consent forms were obtained from the study participants.

## Results

The demographic and clinical characteristics of the patients are presented in Table 1. Of the 37 patients with iris cysts, 24 (65%) were female and 13 (35%) were male. The mean age at diagnosis was 34.4 ± 19.5 (5–85) years. Twenty-one (57%) of the iris cysts were located in the right eye and 16 (43%) in the left eye. The patients were followed for a mean of 21.3 ± 9.6 (median 20) months. Thirty-five (94.5%) of the cysts were classified as primary and 2 (5.5%) as secondary based on etiology. Of the primary cysts, 31 (84%) were pigment epithelial and 4 (11%) were stromal cysts. The secondary cysts were all stromal cysts and developed due to perforating ocular injury. One of these patients was a 5-year-old boy and the other was a 16-year-old girl.

**Figure 1:**
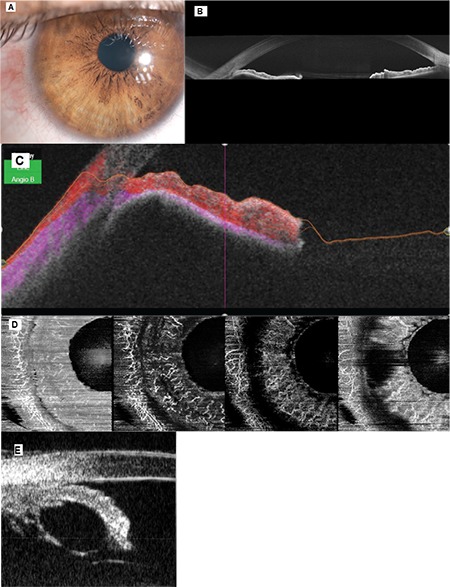
A) Peripheral IPE cyst at 9 o’clock in the right eye with hyperemia in the adjacent conjunctiva. B) Anterior segment SS-OCT image of a temporal iris cyst shows anterior protrusion of the iris, while the posterior borders of the cyst are not clearly distinguishable. C) SS-OCT anterior segment B-scan angiography image of a temporal peripheral iris cyst shows no intrinsic vascularization in the mass causing anterior protrusion of the iris. D) SS-OCTA images of a temporal peripheral IPE cyst from 4 different layers of the iris. A signal void is observed in the cyst region. E) The anterior and posterior walls of a hollow peripheral IPE cyst are clearly visible on UBM

**Figure 2:**
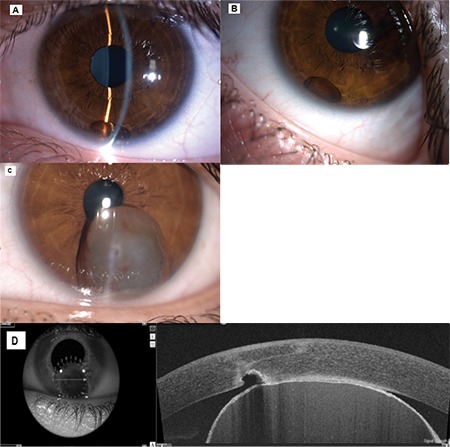
A) A dislodged (free-floating) iris cyst. B) Free movement of the dislodged (free-floating) iris cyst is observed as head position changes. C) Stromal iris cyst secondary to penetrating trauma. D) Anterior segment SS-OCT shows a stromal cyst secondary to trauma with a hyporeflective center and hyperreflective wall touching the corneal endothelium, and a defect in the endothelium/Descemet’s membrane/deep stroma

UBM was performed on 26 patients with iris cysts. On UBM, IPE cysts appeared as thin-walled with no solid lesion component (Figure 1e). According to UBM and clinical findings, 26 (72.2%) of the primary IPE cysts were peripheral (Figure 1a), 4 (11.1%) were midzonal, and 1 was free-floating/dislodged (2.7%) (Figure 2a-b). Patients with peripheral IPE cysts had a mean age of 30.4±10.7 (16-53) and included 18 females and 8 males. Patients with midzonal IPE cysts had a mean age of 52±19.2 (29-72) and included 3 men and 1 woman. The patient with dislodged IPE cyst was a 20-year-old woman. Of the primary stromal cysts, 3 (8.1%) were acquired (Figure 2c) and 1 was congenital (2.7%). These patients had a mean age of 52±33.5 (17-85) years and included 3 females and 1 male. The mean size of pigment epithelial cysts was 1.7x 2.0 mm and that of stromal cysts was 5.2x3.5 mm.

SS-OCT and SS-OCTA were performed on 4 of the patients. AS SS-OCT was used to study iris configuration and the relationship of peripheral IPE cysts to the anterior chamber angle (Figure 1b). AS SS-OCT images of a patient with stromal iris cyst secondary to penetrating trauma by knife revealed a hollow cyst with a thin, hyperreflective wall that had grown to the point of touching the corneal endothelium, as well as a tissue defect on the posterior corneal surface (Figure 2D). The anterior surfaces of the cysts showed high reflectivity but the posterior surface boundaries could not be distinguished. AS SS-OCTA was used to evaluate intrinsic vascularity and blood flow in the cysts. No intrinsic tumoral vascularity or blood flow was observed in any of the cysts (flow void) (Figure 1C, D).

In terms of cyst location, 14 (37.8%) were inferior, 12 (32.4%) were temporal, 8 (21.6%) were nasal, and 3 (8.1%) were superior (4:30-7:30 o’clock was regarded as inferior, 7:30-10:30 o’clock as temporal/nasal, 10:30-1:30 o’clock as superior, and 1:30-4:30 o’clock as the nasal/temporal region). 

As treatment, monitoring was recommended for all patients with pigment epithelial cysts. No growth or intraocular complications were observed in any of the patients. Of the 4 patients with primary stromal cyst, 3 were treated by surgical excision while monitoring was recommended for the other. Surgical resection was performed on both patients with secondary stromal cyst. Three patients underwent surgical resection via partial lamellar sclerouvectomy (PLSU). One of these patients was first treated with cyst aspiration and ethanol injection into the lesion, and resection by PLSU was done later done when recurrence was detected. Two patients underwent surgical resection through a limbal incision. One patient with a congenital stromal cyst who had a limbal incision developed secondary glaucoma and band keratopathy after resection of the 6x5 mm cyst. In final follow-up examination, all patients who were followed by clinical observation and those who underwent surgery were in stable condition.

## Discussion

Peripheral (iridociliary) cysts are the most common type of IPE cysts. Lois et al.^2^ reported that peripheral IPE cysts present more frequently after the age of 33 and in women. In 26 of our patients, IPE cysts were peripheral. Their mean age was 30.4±10.7 years, with 18 being female and 8 male. Although peripheral IPE cysts are common, the diagnosis may be overlooked in clinical practice because they are asymptomatic and located in the iridociliary sulcus.^15^ They are usually noticed during routine slit-lamp examination as an anterior elevation of the iris stroma in the nasal or temporal region (Figure 1A). UBM should be performed to differentiate them from solid tumors. They appear as hollow, thin-walled lesions on UBM.^14,15^

Midzonal IPE cysts are located in the middle region of the iris. They are usually noticed during periodic examination or cataract surgery.They emerge around the age of 52 on average, with no sex difference.^2^ IPE cysts were midzonal in 4 of our patients. Their mean age was 52±19.2 years, with 3 being male and 1 female. Pupillary (central) IPE cysts are located at the pupil margin and may be visible without the need for dilation.

IPE cysts can sometimes dislodge from their initial location and float freely in the anterior chamber or vitreous humor.^18,19^ Dislodged IPE cysts floating freely in the anterior chamber change location according to head position due to gravity. Dislodged cysts in the anterior chamber can sometimes settle in the angle.^15^ Cysts in the vitreous humor, which has a collagen/hyaluronic acid roof, are less mobile. The 1 patient with dislodged iris cyst in our study was a 20-year-old woman (Figure 2A, B). Free-floating pigmented cysts in the vitreous may originate from the IPE or ciliary pigment epithelium. Previous studies have shown that iris cysts floating in the vitreous may have originated from the pigmented ciliary body epithelium in the pars plana region after blunt trauma.^20,21,22,23,24^ Free-floating implantation cysts may cause corneal endothelial damage and reduce endothelial cell count. Therefore, free-floating cysts should be monitored regularly, and intervention should be considered if endothelial damage is observed.

IPE cysts are generally asymptomatic and do not require treatment except in rare cases that affect the visual axis or involve the anterior chamber angle.^2,4,5^ Such cases can be treated with laser ablation or cyst evacuation by needle aspiration.^15^ Surgical resection may be necessary for large cysts that fill the anterior chamber. Follow-up was the recommended treatment approach for all of our patients and none developed clinical complications during the follow-up period. 

Stromal iris cysts are more anteriorly located compared to IPE cysts and can slowly grow to fill the anterior chamber and pupillary region. They may remain the same size for years, or enlarge suddenly and rupture, leading to secondary glaucoma and corneal decompensation. They include congenital and acquired subtypes. Congenital cysts usually appear before the age of 10 and are aggressive.^11,12^ Although spontaneous regression can occur in rare cases, most stromal cysts require treatment by needle aspiration or surgical excision.^11,13^ Four of our patients had stromal cysts; 1 was congenital and 3 were acquired. The congenital cyst was 6x5 mm in size and was treated with surgical resection through a limbal incision. Secondary glaucoma and band keratopathy developed after resection. Of the 3 patients with acquired cysts, 1 was recommended follow-up while 2 were treated with surgical resection by the PLSU method. One of these patients was first treated with cyst aspiration and ethanol injection and underwent resection with PLSU upon detection of recurrence. Leakage into the eye should be avoided during ethanol injection. For this reason, the cyst wall should be carefully perforated with the needle. Care should also be taken not to leave residual cyst tissue during PLSU.

Secondary cysts usually develop after ocular surgery or trauma. They usually form due to surface epithelial cells from the conjunctiva or cornea growing inward and accumulating on the iris after a penetrating or surgical trauma (implantation cysts).^7,19^ Though rare, secondary iris cysts can also results from the prolonged use of miotic drugs and prostaglandins; parasitic infections such as ocular cysticercosis; inflammatory conditions such as uveitis; or medulloepithelioma, melanoma, nevi, and metastatic tumors.^8,9,10,25^ Secondary cysts grow faster than primary cysts and as a result may lead to complications such as uveitis, decreased vision, secondary glaucoma, lens subluxation, iris bombe, or complicated cataract. Two of our patients had secondary cysts associated with perforating ocular injury (Figure 2C). Both underwent surgical resection, one with the PLSU method and the other through a limbal incision.

Imaging methods are important in the differential diagnosis of cysts and ocular tumors. Melanoma and pigment epithelial adenoma should be ruled out in patients with IPE cysts. IPE cysts appear as thin-walled, hollow, and homogenous lesions with regular borders on UBM and AS OCT, while tumors present a solid inner structure.^14,26,27^ In the differential diagnosis from iris melanoma, the main indicator of malignancy was shown to be increase in lesion size.^28,29^ UBM is used to evaluate lesion growth by objectively measuring the maximum thickness and diameter of the iris lesion (Figure 1E). It provides valuable information about the AS due to its high penetration power, ability to show ciliary body extension, and because it is unaffected by degree of pigmentation and enables good visualization of the posterior border of the tumor.^30^

Though rare, cyst-like cavitary spaces appearing on ultrasound as hollow lesions are found in cases of uveal melanoma.^31,32^ In such cases of cavitary melanoma, is important to make a differential diagnosis between solid and cystic lesions. Cavitary lesions have much thicker walls than cysts.

AS OCT is a noncontact imaging method that provides high-resolution cross-sectional images and is used in the imaging of many pathological conditions such as iris cyst, iris nevus, and iris/ciliary body melanoma. This method may be unable to show the posterior wall of the cyst due to the absorption of light by the IPE layer, especially in dark-colored eyes. Bianciotto et al.^33^ compared UBM and AS OCT in a series of 200 AS tumors and demonstrated that AS OCT images had lower resolution compared to UBM due to the shadowing effect in large or pigmented lesions originating from the IPE or ciliary body, and that UBM was superior to AS OCT in displaying the posterior lesion border and tumor configuration. Previous studies have also shown UBM to be superior to AS OCT in the evaluation and follow-up of AS tumors due to its effective visualization of large, pigmented tumors and ciliary body tumors.^26,34,35,36^ In the case of our 4 patients who underwent AS SS-OCT imaging, the internal structure of the lesions was analyzed by measuring base diameter and thickness. The AS SS-OCT image of a patient with iris cyst secondary to penetrating knife trauma revealed a hollow cyst with a thin, hyperreflective wall that had grown and touched the corneal endothelium, in addition to a tissue defect on the posterior surface of the cornea (Figure 2D). While the anterior surfaces of IPE cysts were visible on AS SS-OCT as high reflectivity, the posterior borders could not be distinguished (Figure 1B).

OCTA is a noninvasive imaging method based on an algorithm-based evaluation of changes in the intensity and phase of light reflected by the movement of red blood cells within a vessel. This method enables the detailed visualization of the retinal and choroidal vascular networks, has recently been used for the examination of AS tumors. The visibility of the vascular structures in the iris varies depending on pigment density. Consistent with this, intrinsic intratumoral vascularity and vascular flow were not observed in any of the 4 patients in our study who underwent SS-OCTA (Figure 1C, D). These findings were found to be important in the differentiation of cystic and solid tumors.^3^

### Study Limitations

The main limitation of this study is that due to the small patient number, we were unable to analyze pupillary primary IPE cysts, which are rarer in clinical practice.

## Conclusion

In conclusion, cystic lesions of the iris originate from the pigment epithelium or stroma. Most cysts are of primary etiology, pigmentary epithelial origin, and are located peripherally. Pigment epithelial cysts do not require any treatment, whereas iris stromal cysts usually require treatment. Although both UBM and AS OCT play an important role in diagnosis and treatment follow-up of iris cysts, UBM is superior to AS OCT in the imaging of iris lesions and the differentiation of cystic and solid lesions. With the development of AS OCTA techniques, it is also possible to noninvasively obtain information about the internal vascular structure of these tumors.

## Figures and Tables

**Table 1:**
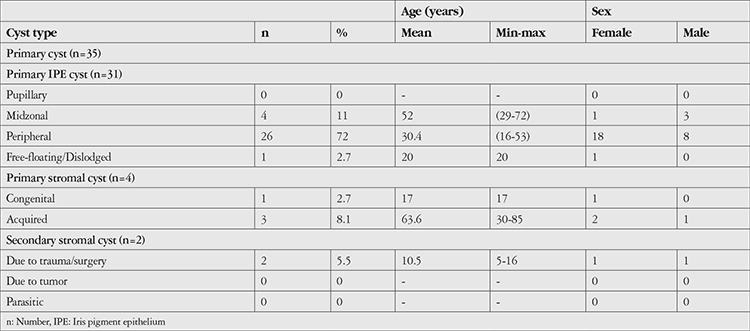
Clinical and demographic characteristics of the 37 patients with iris cyst
